# Bound States in the Continuum in double layer structures

**DOI:** 10.1038/srep26988

**Published:** 2016-06-01

**Authors:** LiangSheng Li, Hongcheng Yin

**Affiliations:** 1 Science and Technology on Electromagnetic Scattering Laboratory, Beijing 100854, China

## Abstract

We have theoretically investigated the reflectivity spectrums of single- and double-layer photonic crystal slabs and the dielectric multilayer stack. It is shown that light can be perfectly confined in a single-layer photonic crystal slab at a given incident angle by changing the thickness, permittivity or hole radius of the structure. With a tunable double-layer photonic crystal slab, we demonstrate that the occurrence of tunable bound states in the continuum is dependent on the spacing between two slabs. Moreover, by analytically investigating the Drude lossless multilayer stack model, the spacing dependence of bound states in the continuum is characterized as the phase matching condition that illuminates these states can occur at any nonzero incident angles by adjusting the spacing.

Photonic crystal (PhC) slabs with a periodic modulation of refractive index are used in many applications including radiation modulation^1^, cavity photon resonator^2^,^3^, biosensing^4^ and polarization selectivity^5^. The periodic modulation alters the dispersion relation of light in the slabs and gives rise to Fano resonances originating from the coupling between the guided modes supported by the slab and external plane waves^6^. When the linewidth of Fano resonaces tends to zero, the light is perfectly confined in the slab, and one can observe the bound states in the continuum (BICs), which were first discovered by von Neumann and Wigner in quantum systems^7^ and interpreted as the interference of quasi-stationary states between direct and via-the-continuum channels^8^. BICs can be shown in various systems, because the existence of topological charges is defined by the winding number of the polarization vectors around the BIC^9^. These robust states in photonics can occur as results of different mechanisms, such as the incompatible symmetry^10^,^11^,^12^,^13^, the zero refraction index^14^,^15^, the Fabry-Perot resonance^16^,^17^,^18^ or the destructive interference^19^,^20^. This specific resonance state has a high quality factor making it potentially useful for filters, antennas, chemical sensing, or single-photon sources^21^,^22^,^23^,^24^,^25^. However, all reported systems have BICs at an uncontrollable incident angle. A fundamental question is thus to know whether there exist structures in which BICs can be exhibited at a designed incident angle.

In this paper, we can observe BICs at a given incident angle to vary the materials parameter of PhC slabs. It is noted, however, that experimental observation of these BICs is difficult and even could not be realized in practice. In order to overcome this technical obstacle, we use a mechanically tunable double layer structure consisting of coupled PhC slabs to produce the BICs at an expectable angle. These results make an improvement to apply BICs to the many sensing situations requiring both high-quality factor and strong light matter interaction at a given incident angle.

First, we consider a photonic crystal slab with finite thickness *L* (Fig. 1a inset), where a square lattice (periodicity *W*) of circular air holes (diameter D). This single layer PhC slab structure has an inversion geometric symmetry and a mirror geometric symmetry in the z direction. Light incident on the single layer PhC slab can excite the Fano resonances observed in the reflectivity spectrum due to interference between in-plane waveguide modes and out-of-plane far-field radiation^6^. Figure 1(a) shows that Fano resonances are characterized by the asymmetric profile consisted of blue and red branches corresponding to the reflectivity of zero and one, respectively. When thickness, hole diameter, relative permittivity (*ε*), incident angle (*θ*) and incident frequency (*ω*) are suitable, the blue and red branches meet each other and produce a BIC with vanishing resonance width^19^. In the region one of this contour plot, a fixed BIC occurs at *θ* = 0 because the geometric symmetry forbids coupling to any far-field radiation^11^. When *θ* ≠ 0, the appearance of a tunable BIC, in the region two, may be understood as the weighted destructive interference between the waveguide modes and radiating field via the accidental triangle symmetry of in-plane wave vectors^10^. Because the requirement of weighted destructive interference depends on the properties of PhC slab, the occurrence of tunable BICs may be designed and manipulated for any nonzero incident angles. For an example, when *θ* = *π*/6, two new BICs, in the region 3 and 4, occur by adjusting the thickness of slab as shown in Fig. 1(b). Furthermore, a new BIC in the region 5 can be achieved by the modification of relative permittivity of slab, as shown in Fig. 1(c). As a result, for any nonzero incident angles, the tunable BICs can always be produced by changing thickness and relative permittivity. Figure 1(d) show a trivial way 6 to find a BIC in the region by decreasing the hole radius.

One interesting question thus is to conveniently generate BICs for any nonzero incident angles. Here, we consider a mechanically tunable double layer structure consisting of coupled PhC slabs^25^,^26^,^27^,^28^, which keeps the geometric symmetry and has a controllable spacing between two slabs, as shown in Fig. 2(a). Each PhC slab has a fixed finite thickness, periodicity and hole diameter. When the spacing (h) between slabs is finely adjusted for various incident angles, the sharp Fano resonance features are clearly visible and the tunable BICs are observed shown in Fig. 2(b–f). In this double layer structure as mirrors to form an optical cavity, the interaction between the resonances localized on the each slab is highly sensitive to the spacing *h*^16^. Therefore, the h-dependence interaction results in the h-dependence weighted destructive interference, which indicates that the Fabry-Perot–type resonance plays an important role in the formation of BICs. By changing the displacement between slabs, we can capture the BICs at an arbitrarily incident angle.

In order to gain further physical insights of the BIC in the double layer structure, we plot in Fig. 3(a) quality factor and in Fig. 3(b) the distributions of the electric field intensity in xy-plane at the resonance frequency *ω* = 499.73(THz) with various z. The Fano resonance here has a sharp asymmetrical profile with extremely large, but finite, quality factor. The distribution of field intensity becomes more localized inside the double layer structure by the formation of BICs, and the outside field tends to vanish. To better understand the BIC of the double layer structure consisting of coupled PhC slabs, the effective permittivity is plotted in Fig. 4(a). It can be observed that the effective zero-permittivity corresponds to the maximum of reflectivity shown in Fig. 4(b). Thus, in the vicinity of the resonances, the PhC layer can be understood by the effective media, which has a zero-permittivity at a special frequency.

In order to understand the h-dependence of BICs, we consider that the dielectric multilayer stack, displayed in Fig. 5(a), is made of three nonmagnetic dielectric layers with a permittivity of *ε*_1_, *ε*_2_ or *ε*_3_ and a thickness of *L*_1_, *L*_2_ or *L*_3_ surrounded by the materials *ε*_*in*_ and *ε*_*out*_. Reflectivity for p-polarized light can be easily derived from the transfer matrix method and written as


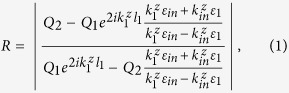


here, and the coefficients are given by


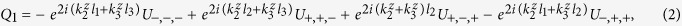



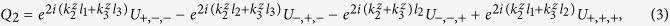


where 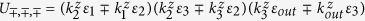, 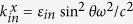
is the x-direction wave number, *θ* is the incident angle, *c* is the speed of light in a vacuum, 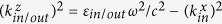, and 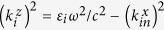
are the z-direction wave number in the surrounded material and medium *i* = 1, 2, 3, 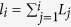 is the position of interface *j* = 1, 2, 3. Then, we assume that the geometrical symmetrical sandwich structure with the lossless Drude relative permittivity 
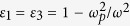
 and the thickness *L*_1_ = *L*_3_ is surrounded by the vacuum *ε*_*in*_ = *ε*_*out*_ = 1, where *ω*_*p*_ is plasma frequency. To observe BICs for such a sandwich structure, we plot the reflectivity spectrum, computed by the exact analytic theory, as a function of incident angle and frequency in Fig. 3(b). When *ω* = *ω*_*p*_, the top and bottom layers have zero-permittivity and reflectivity is equal to one. When 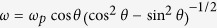, reflectivity is equal to zero owing to destructive interference. The relative width of Fano resonances is defined by


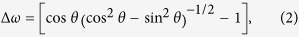


which only depends on the incident angle and elucidate that the red branches and blue branches meet each other to produce a fixed BIC at *θ* = 0. The occurrence of tunable BICs in symmetrical sandwich structures requires the top and bottom layers to have permittivity equal to zero and the phase matching condition is satisfied as


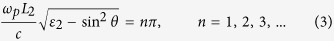


This condition illuminates that the appearance of BICs is dependent on the thickness and permittivity of the middle layer, plasma frequency, and incident angle. It should be emphasized that the thickness of middle layer, here, plays a similar role as the spacing h between two PhC slabs in the formation of tunable BICs. In the example of Fig. 5(b), the requirements of tunable BICs are met when *θ* = arc cos (2π/7.2) analytically calculated from Eq. (2).

When *ε*_2_ = 1, *L*_1_ = 100 nm, ω_*p*_ = 3×10^14^ Hz, and *θ* = π/6, the corresponding reflectivity is plotted as a function of ratio *L*_2_/*L*_1_ at various incident frequencies as displayed in Fig. 6(a). From the Eq. (2), we precisely know that the requirement of BICs is satisfied when the ratio between thicknesses is *L*_2_/*L*_1_ = *np*20/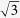. The result demonstrates that it is indeed possible to produce a tunable BIC for any incident angle by changing the thickness of middle layer (gap between two slabs). When L_2_ = 1900 nm and *ε*_2_ = *n*^2^π^2^/3.61 + 1/4, the phase matching condition is met and a series of BICs is shown in Fig. 6(b).

The asymmetrical profile of the reflectivity has maximum (+) and minimum (−) as shown in Fig. 7(a). Figure 7(b) shows that the positions of the maximum and minimum are a function of the reduced thickness. When the curves intersect, the width of the resonance becomes zero corresponding to a BIC. The scattering eigenmodes of the system can be observed by using the singularity of coefficient matrix M


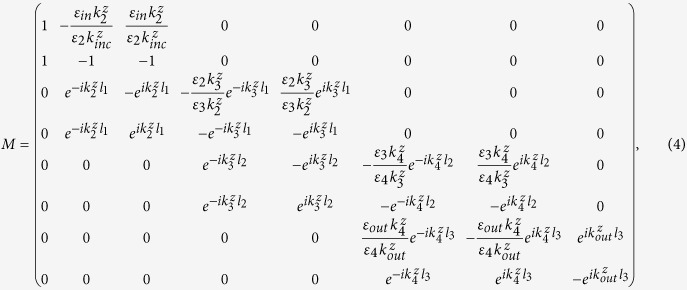


where *l*_1_ = *L*_1_, *l*_2_ = *L*_1_ + *L*_2_, *l*_3_ = *L*_1_ + *L*_2_ + *L*_3_. In Fig. 7(c,d), we plot real (*ω*_*r*_) and imaginary (*ω*_*i*_) parts of scattering eigenmodes of the linear system. When *ω*_*i*_ = 0, the eigenfrequency becomes purely real and the lifetime of the Fano resonance diverges. The quality factors of the resonator is defined by *Q*_*f*_ = *ω*_*r*_/(−2*ω*_*i*_) as shown in Fig. 7(e). Infinity quality factors of resonance for various incident angles are clearly visible, while the positions of BIC occurrence can be predicted by Eq. (3).

In conclusion, we have shown that the tunable BICs can be easily achieved at any incident angle by adjusting the distance between PhC slabs due to weight destructive interferences without resorting to change thickness, permittivity or hole radius of slabs. The resonance behaviors of the PhC slab can be understood by the effective media, which has a zero-permittivity at a special frequency. Those results suggest that at any incident angle the occurrence of tunable BICs in the double PhC layer structure can be understood by the effective zero-permittivity materials. Therefore, the symmetrical sandwich structure described by lossless Drude model is introduced to reproduce the BICs behavior including its dependence on distance between two slabs. An analytical perspective for h-dependence of BICs is provided to demonstrate that the BICs occur through the new phase matching condition. The double-slab structure described here provides a simple platform for studying the interacting resonance between slabs.

## Additional Information

**How to cite this article**: Li, L. and Yin, H. Bound States in the Continuum in double layer structures. *Sci. Rep*. **6**, 26988; doi: 10.1038/srep26988 (2016).

## Figures and Tables

**Figure 1:**
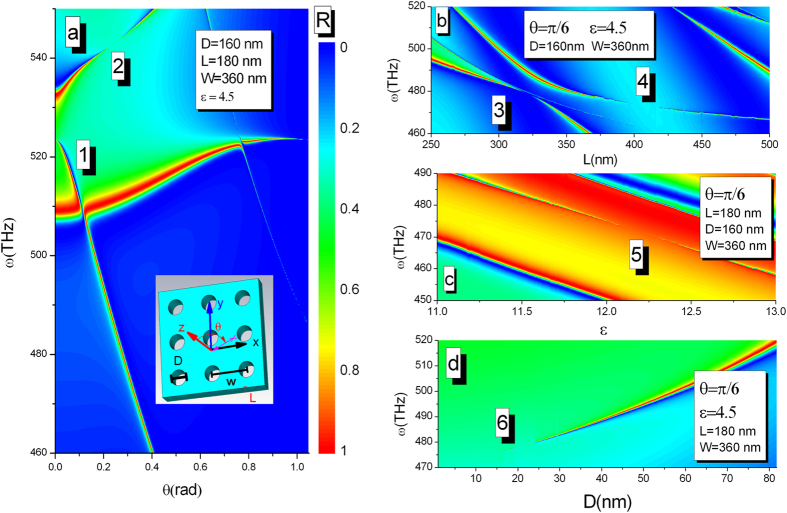
(**a**) Numerical reflectivity spectra (R) for the p-polarized as a function of incident angle and frequency. Numbers within the plots indicate the regions where a BIC occurs. The inset shows a schematic photonic crystal structure. For a given incident angle, the reflectivity spectrum for p-polarized light as a function of frequency and thickness (**b**), relative permittivity (**c**), or hole diameter (**d**).

**Figure 2:**
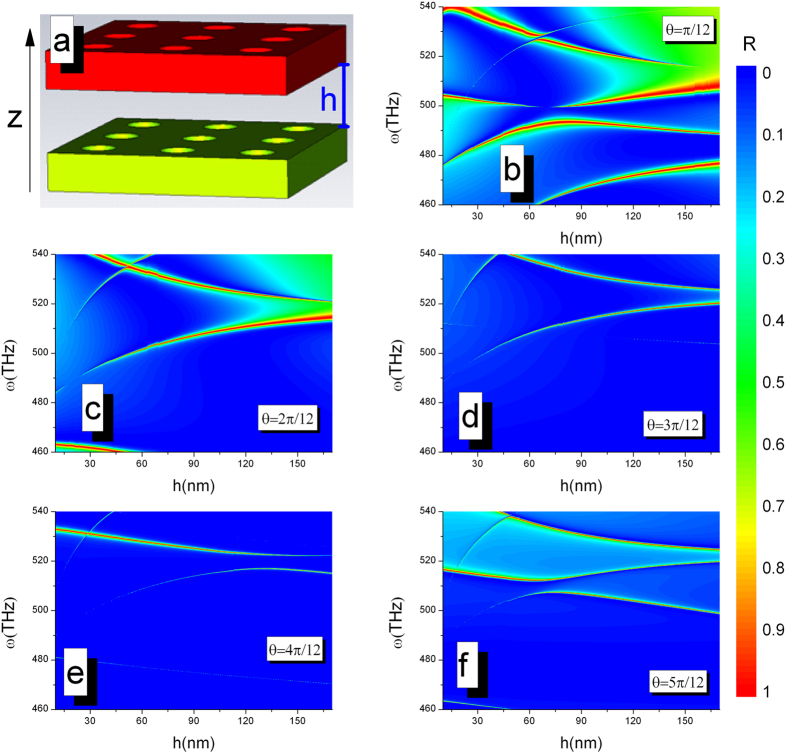
(**a**) Schematic of a tunable double layer structure consisting of coupled PhC slabs. The black arrow represents the z-direction. The single PhC slab: thickness *L* = 180 *nm*, periodicity *W* = 360 *nm*, hole diameter *D* = 160 *nm*, and relative permittivity *ε* = 4.5. Reflectivity spectra of p-polarized light are influenced by the spacing *h* between the slabs for the various value of incident angle (**b**) *θ* = *π*/12, (**c**) *θ* = 2*π*/12, (**d**) *θ* = 3*π*/12, (**e**) *θ* = 4*π*/12, and (**f** ) *θ* = 5*π*/12.

**Figure 3:**
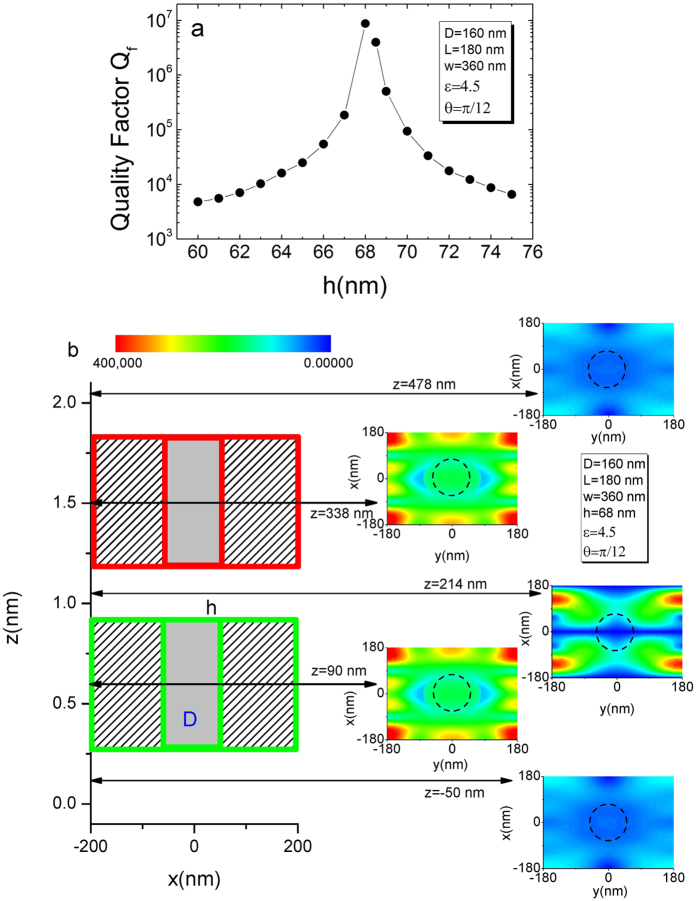
(**a**) Quality factor of two PhC slabs as a function of gap h. (**b**) The green (red) box indicate a PhC slab. The regions of gray color represent the holes in the slabs. Right: slices at five representative positions. Spatial distribution of the power density in electric fields on z-direction slices at the resonance frequency *ω* = 499.73(THz) with various z.

**Figure 4:**
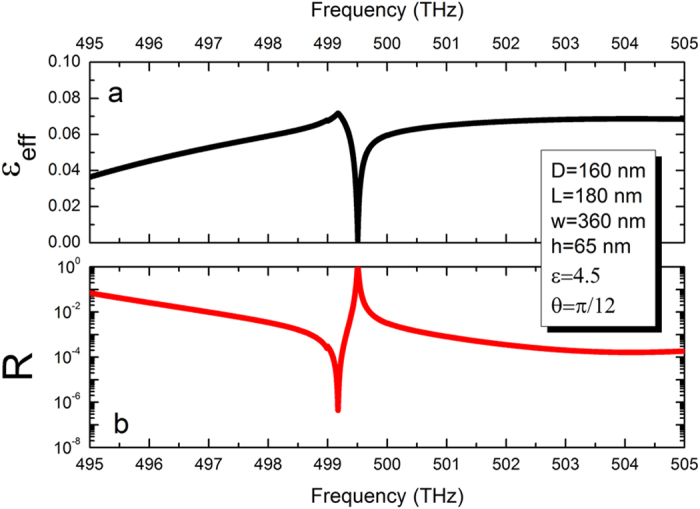
(**a**) Extracted effective permittivity of the PhC slab as a function of the incident frequency calculated using a numerical retrieval of the effective permittivity. (**b**) Reflectivity as a function of the incident frequency.

**Figure 5:**
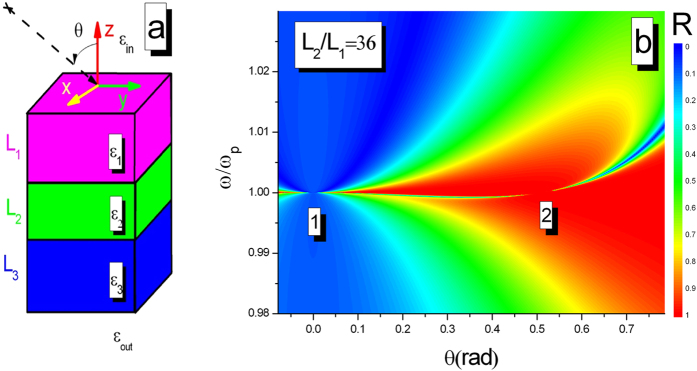
(**a**) Schematics of a multilayer structure surrounded by materials with permittivities *ε*_*in*_ and *ε*_*out*_. Light incident on the slab at angle *θ*. (**b**) Reflectivity Spectrum for p-polarized light as a function of the incident frequency and angle when *ε*_2_ = 1, *L*_1_ = *L*_3_ = 100 nm, *L*_2_ = 3600 nm and *ω*_*p*_ = 3 × 10^14^ Hz. Numbers within the plots indicate the regions where a BIC occurs.

**Figure 6:**
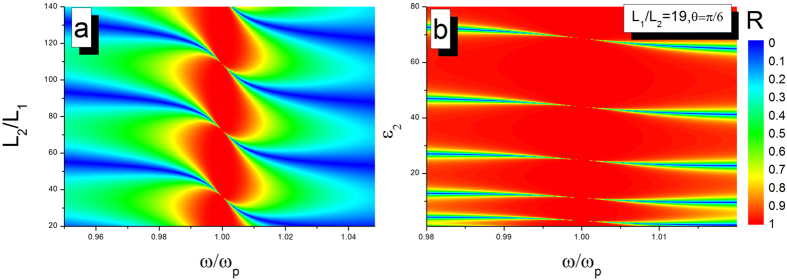
Reflectivity spectra for p-polarized light as a function of the incident frequency and reduced thickness *L*_2_/*L*_1_ (**a**) or permittivity (**b**).

**Figure 7:**
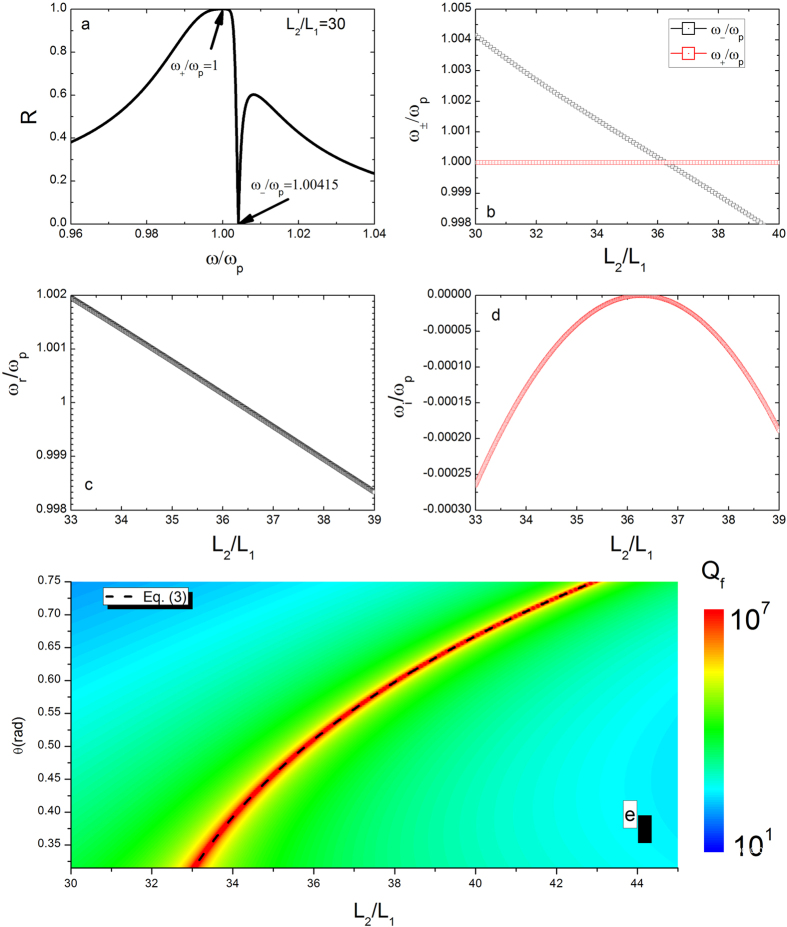
(**a**) Reflectivity as a function of the incident frequency. (**b**) The positions of the maximum (+) and minimum (−) versus the reduced thickness. (**c**) Real and (**d**) imaginary parts of oscillation frequency of the linear system as a function of reduced thickness. (**e**) Quality factor as a function of the reduced thickness and angle when *ε*_2_ = 1, *L*_1_ = *L*_3_ = 100 nm, *L*_2_ = 3600 nm and *ω*_*p*_ = 3 × 10^14^ Hz. Eq. (3) is plotted as a dashed line, demonstrating the positions of infinity quality factor.
